# Associations of Calcium from Food Sources versus Phosphate Binders with Serum Calcium and FGF23 in Hemodialysis Patients

**DOI:** 10.3390/jcm8101680

**Published:** 2019-10-14

**Authors:** Sara Mahdavi, Antonio Bellasi, Karan Nagra, Luke Johnston, Paul Tam, Biagio Di Iorio, Tabo Sikaneta

**Affiliations:** 1 Department of Nutritional Sciences, University of Toronto, Toronto, ON M5S 1A8, Canada; smahdavi.rd@gmail.com (S.M.); luke.johnston@mail.utoronto.ca (L.J.); 2 Department of Nephrology, The Scarborough Hospital, Scarborough, ON M1P 2V5, Canada; pywtam@gmail.com; 3 Family and Community Medicine, University of Toronto, Toronto, ON M5G 1V7, Canada; knagra@ualberta.ca; 4 Department of Research, Innovation and Brand Reputation, ASST Papa Giovanni XXIII, 24127 Bergamo, Italy; abellasi@asst-pg23.it; 5 Department of Medicine, AORN “Antonio Cardarelli”, 80231 Neaples, Italy; br.diiorio@gmail.com

**Keywords:** fibroblast growth factor 23 (FGF23), calcium balance, dietary calcium, calcium supplements, serum calcium

## Abstract

Background: Dysregulated serum calcium and FGF23 are associated with increased mortality and morbidity rates in patients receiving hemodialysis. Preliminary data suggest serum calcium regulates FGF23 secretion independently of serum phosphate, parathyroid hormone, and 25-OH vitamin D. It is unclear to what extent dietary and prescription sources of calcium influence calcium and FGF23 levels, and whether they confound this relationship. In this cross-sectional analysis of a multi-ethnic cohort of prevalent hemodialysis patients, association of dietary calcium and prescribed calcium were examined against serum calcium and FGF23. Bi- and multivariable linear regression was used for all analyses. Results: 81 patients (mean age 58 years, dialysis vintage 2 years, 51 men) participated. Dietary calcium was inversely associated with FGF23 (*p* = 0.04) however association of FGF23 with prescribed calcium did not reach statistical significance (0.08). In multivariable models, dietary calcium and prescribed calcium were associated in opposing directions with serum calcium (prescribed calcium; ß-coefficient = −0.35, *p* = 0.005 versus dietary calcium; ß-coefficient = 0.35, *p* = 0.03). FGF23 was independently associated with serum calcium (*p* = 0.007). Conclusions: We found differing, sometimes opposing, associations between serum calcium and FGF23 levels when considering prescribed versus dietary sources of calcium. Serum calcium and FGF23 were strongly correlated regardless of possible confounders examined in this hemodialysis cohort. Dietary calcium was associated with higher serum calcium and lower FGF23 concentrations, while prescribed calcium was only inversely associated with serum calcium. Further studies are required to confirm these associations and determine causality.

## 1. Introduction

Abnormal serum concentrations of calcium and fibroblast growth factor 23 (FGF23) are two of a group of interrelated metabolic disturbances—collectively referred to as the chronic kidney disease-mineral bone disorder (CKD-MBD)—that are implicated in the high morbidity and mortality rates in CKD. Hypocalcemia predicts increased rates of progression of CKD and left ventricular hypertrophy. Hypercalcemia is implicated in vascular and valvular calcification, and elevated serum FGF23 is a clear marker for increased cardiovascular and overall mortality [1,2]. Serum calcium and FGF23 correlate with each other independently of serum 25-OH vitamin D, parathyroid hormone (PTH), and serum phosphate levels in what appears to be a negative feedback loop [3,4]. However, serum calcium and FGF23 concentrations are differentially altered by other factors that may confound this association. These confounders and association have not often been accounted for in previous research studies. Clinicians treating CKD-MBD manipulate dietary phosphate and prescribed calcium-based-phosphate binders in effort to offset serum-mineral dysregulation in hemodialysis patients. However, not all factors are accounted for in these groups. For example; prescribed calcium is associated with higher serum calcium but unchanged serum FGF23, while dietary phosphate is associated with FGF23 but inversely correlated with serum calcium and the impact of dietary calcium with serum calcium and FGF23 remains entirely unexplored [5,6,7].

In the present study, we sought to confirm a correlation between serum calcium and FGF23 in a multi-ethnic cohort of patients receiving hemodialysis in Toronto, Canada. We then determined whether and how prescribed calcium-based phosphate binders versus dietary calcium were associated with serum calcium and FGF23.

## 2. Methods

### 2.1. Ethical Statement

i. Compliance with ethical standards—study procedures were conducted in accordance with the Good Clinical Practice and the Declaration of Helsinki on biomedical research involving human subjects.

ii. Funding—there was no funding required or obtained for the conduct of this study.

iii. Conflict of interest—the authors have no conflicts of interest to declare.

iv. Ethical approval—written approval to conduct this study was obtained from the Ethics Review Boards of the University of Toronto and The Scarborough Hospital. Original approval at The Scarborough Hospital (study number NEPH49) was issued in September 2010 and a second (study number NEPH59) in November 2015.

v. Informed consent—all patients provided informed consent before participation.

### 2.2. Selection Criteria and Study Design

Eligible subjects were independently ambulatory adults (minimum 18 years of age) receiving thrice-weekly hemodialysis for at least six months at one of two outpatient satellite clinics in Toronto, Canada. They were excluded if hospitalized for any reason within the last 30 days. Study questions were answered using a cross-sectional design.

### 2.3. Outcome Measurements

Demographic variables and clinical history were extracted from chart review. Data concerning prescribed calcium-based phosphate binder therapy were obtained and averaged for 3 months prior to laboratory testing. Subjects completed a 3-day diet record (on a dialysis day, non-dialysis weekend day, and a non-dialysis day during the week). Dietary records were reviewed for accuracy by the study renal dietitian before entry into a diet analysis program (Axxya Nutritionist Pro™, Version 2.5, 2006, Stafford, TX, USA). Nutritional status and overall well-being was assessed with a Malnutrition Inflammation Score (MIS) validated in dialysis patients [8]. Prescribed dose of activated vitamin D (oral and intravenous) was summed and converted to a daily dose. Patients were asked if they were compliant to their prescribed calcium dose and if they were taking supplemental vitamin D or calcium in addition to that prescribed. All oral sources of calcium and vitamin D were accounted for in study data. Blood samples for serum calcium, phosphate, PTH, and 25-OH vitamin D determinations were collected before the second weekly dialysis session as per standard hospital procedures. Patient sera were batched, frozen at −20 ℃, and sent to a reference laboratory for measurement of carboxy-terminal FGF23 levels (sandwich ELISA assay, Immutopics, Inc., San Clemente, CA, USA). This assay measures both C-terminal fragment and intact FGF23 concentrations. 

### 2.4. Statistical Analysis

Data were analyzed in STATA (Stata Statistical Software: Release 13. College Station, TX USA: StataCorp LP). FGF23 was used as measured, and after being normalized by transformation to the natural log form (lnFGF23). LnFGF23 was used for all multivariable analyses. PTH was categorized as less than 10, 10–49, 50–149, 150–249, or ≥250 pmol/L. Linear regression was employed for all bivariable and multivariable analyses. Calcium intake (prescribed and dietary), vitamin D intake (supplemental and dietary, normalized by transformation to natural log form), dialysis vintage, serum phosphate, 25-OH vitamin D, parathyroid hormone (as categorized), hemoglobin, ferritin (normalized by transformation to natural log form), transferrin saturation (normalized to natural log form), nutritional status, age, ethnicity (blacks versus other), and gender were independent variables examined in bivariable analyses. 

Two sets of multivariable linear regression models were created to determine the independent predictors for both serum calcium and lnFGF23. The first set was built from the six most statistically significant results from the respective bivariable analyses, and the second from the results of bivariable analyses, the variables regarding dietary and prescribed factors hemodialysis clinicians interested in, and variables as determined by literature review and the Akaike information criteria [9]. Prescribed calcium and dietary calcium were further transformed to 1000 mg-dose increments to improve data translation for clinician utility. Statistical significance was defined as *p*-value of <0.05. 

## 3. Results

Data were collected from all 81 patients in October 2011. Demographic and clinical variables are presented in Table 1. Fifty-nine (73%) were prescribed calcium-based phosphate binders. Calcium carbonate was the only calcium-based phosphate binder prescribed. None of the patients reported taking calcium supplements, while 19 (24%) took vitamin D supplements. The group was well-nourished with a median Malnutrition Inflammation Score of 5 out of 30 [8]. 

### 3.1. Bivariable Analyses

**Figure 1:**
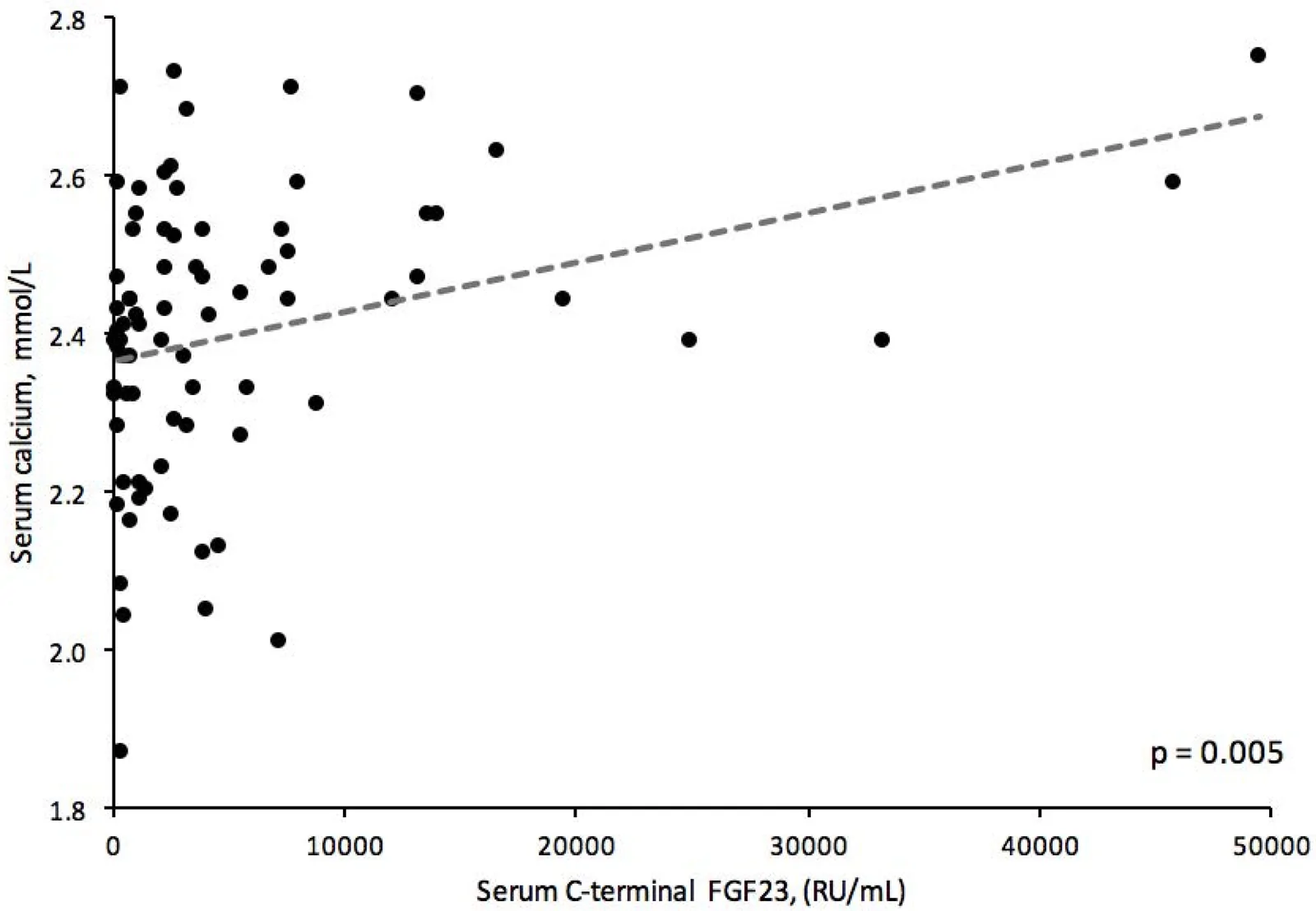
Serum calcium versus FGF23.

**Figure 2:**
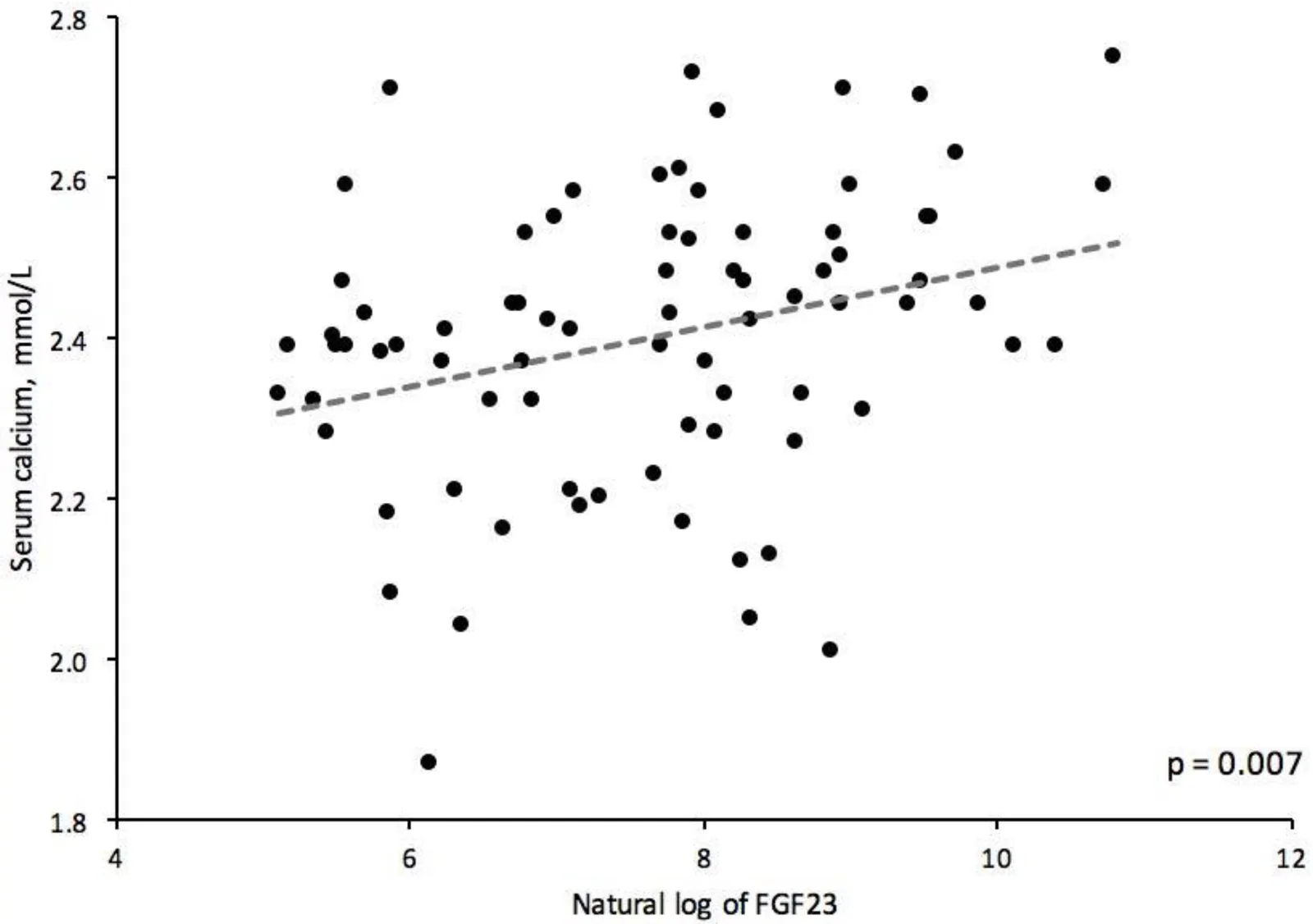
Serum calcium versus the natural log of FGF23.

Bivariable associations of serum calcium and FGF23 are listed in order of statistical significance in Table 2 and Table 3. Serum calcium was associated with FGF23, whether as measured or natural log form (Figure 1 and Figure 2). Serum calcium was also associated with female gender, while FGF23 was also associated with serum phosphate, PTH, and inversely associated with hemoglobin and dietary calcium. 

### 3.2. Multivariable Models

**Figure 3:**
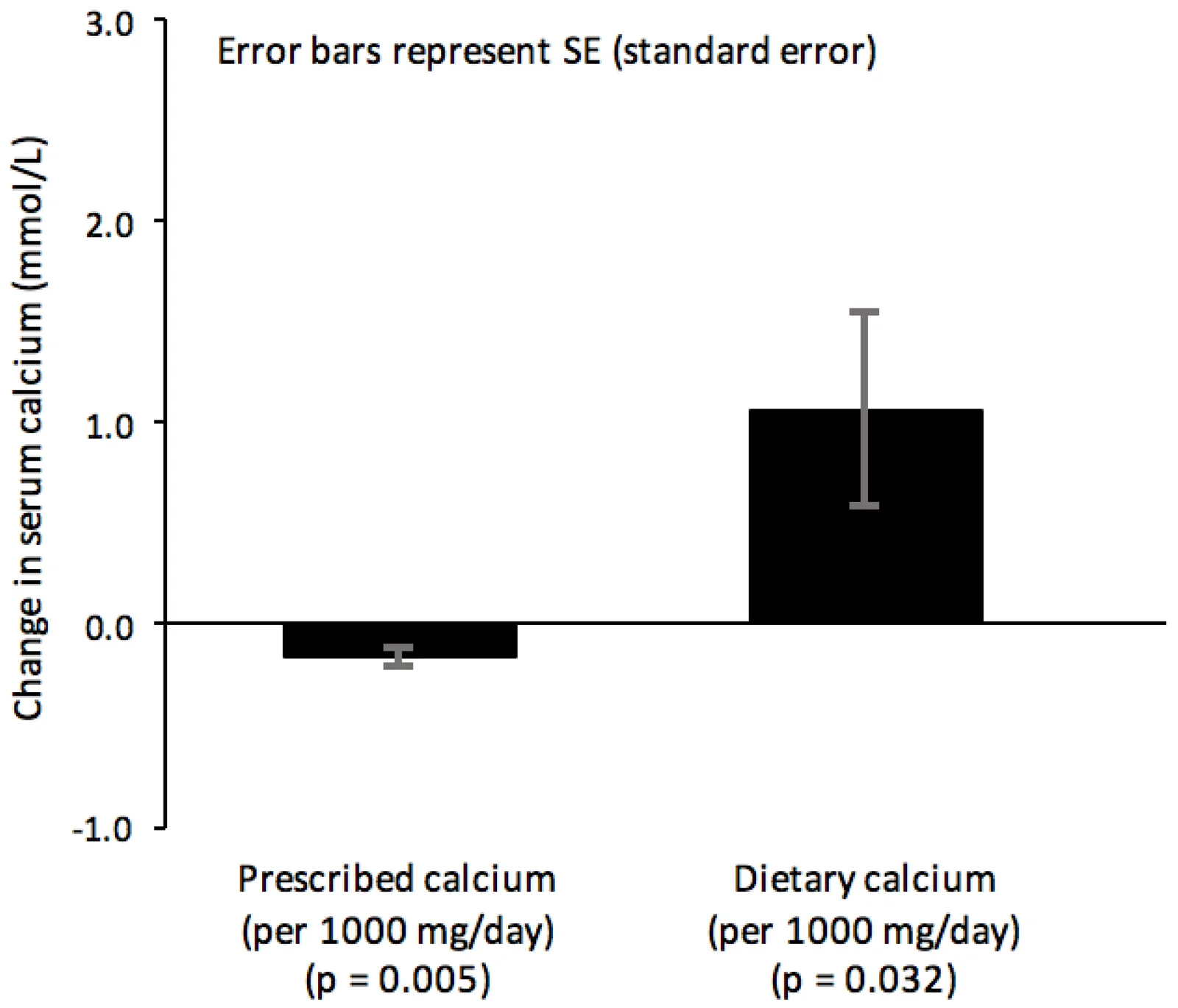
Changes in serum calcium as a function of sources of calcium.

**Figure 4:**
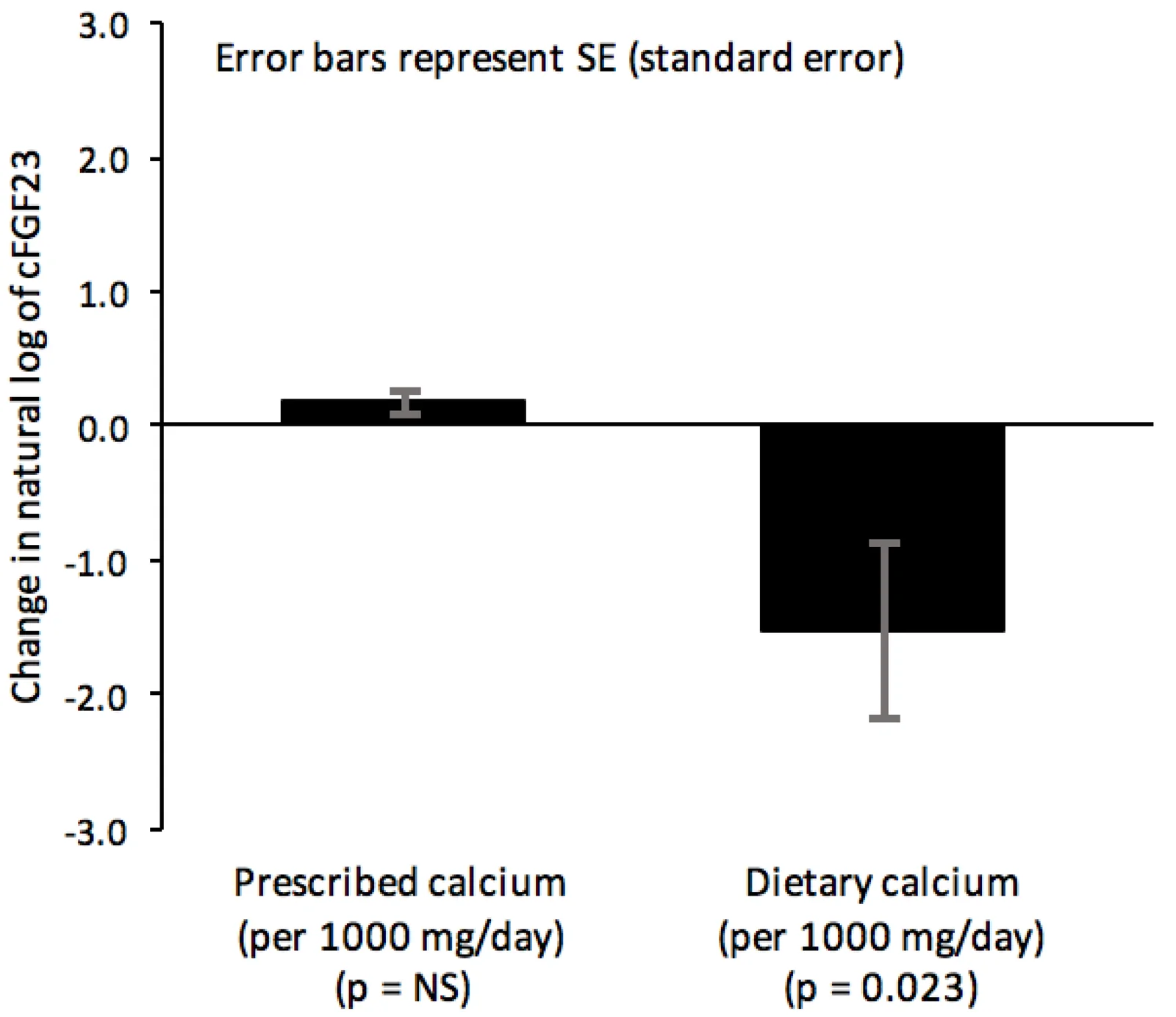
Changes in natural log of FGF23 as a function of sources of calcium.

The results of the first set of models, comprised of the six most significant variables from the respective bivariable analyses, are presented in Table 4 and Table 5. The hand-built models, which improved the power to predict serum calcium and FGF23, are presented in Table 6 and Table 7. Dietary calcium, serum calcium, and hemoglobin were associated with FGF23 in bivariable and both multivariable models, while only FGF23 associated with serum calcium in all examined models. Figure 3 and Figure 4 present associations of dietary vs prescribed calcium with serum calcium and FGF23 (taken from Table 6 and Table 7).

## 4. Discussion

In 2004, Imanishi et al. were the first to report on a correlation between serum calcium and carboxy-terminal FGF23 in a cross-sectional analysis of 158 Japanese males receiving long-term hemodialysis (mean age 61.9 ± 0.9 years, mean dialysis vintage 6.6 years) [3]. This finding has since also been demonstrated in other patients on dialysis, as well as those with primary hyperparathyroidism and tumoral hypercalcemia [10,11,12]. We now confirm these findings in another hemodialysis cohort after adjustments for prescribed calcium and dietary calcium, and other potential confounders. These sources of calcium had different and sometimes opposing associations with serum calcium and FGF23.

FGF23 is a potent risk factor for overall and cardiovascular mortality, and interacts with several factors of importance to the regulation of serum calcium [13]. It participates in a negative feedback loop with 25-OH vitamin D being stimulated by and suppressing its levels. FGF23 also interacts with serum PTH, although the association is less clear and may be based on hypercalcemia [11]. Calcium binds to the FGF23 promoter and increases FGF23 production independently of vitamin D or PTH status in mice with normal renal function [13]. FGF23 production in rats is blunted (even in the face of hyperphosphatemia or hyperparathyroidism) if serum calcium concentrations are below a minimum threshold [4]. Our finding that dietary calcium is associated with rising serum calcium but lower FGF23 levels is difficult to reconcile with these observations, and suggests the possibility that dietary calcium suppresses FGF23 production in hemodialysis patients via a mechanism that is independent of serum calcium levels. Alternatively, a bimodal association of serum calcium and FGF23 may be speculated, where both hypo- and hypercalcemia may be associated with low FGF23 production. The relatively small number of patients in this cohort with hypo- and hypercalcemia prevented us from testing this hypothesis.

The association between FGF23 and serum calcium could conceivably result from increased calcium-based phosphate binder use (hyperphosphatemic patients are more likely to be treated with calcium-based phosphate binders leading to elevated serum calcium). However, while we found a tight correlation between FGF23 and serum phosphate, we did not find an association between CALCIUM-based phosphate binders use and FGF23, and only a weakly negative association between calcium-based phosphate binders and serum calcium. Furthermore, when the interaction term of serum phosphate and prescribed calcium was added to the multivariable model predicting serum calcium, prescribed calcium (but not FGF23) lost statistical significance. If confirmed, these findings suggest that calcium levels are not an accurate marker of calcium-based phosphate binders use, and the reported differential effect of calcium-free vs. calcium-containing phosphate binders on FGF23 is not mediated by serum calcium [6].

Our multivariable models suggest that dietary calcium may be associated with increased calcium and decreased FGF23, and prescribed calcium with decreased calcium and a non-significant trend to increased FGF23 concentrations. Other studies to examine the relative effects of dietary versus prescribed calcium also support different biological effects [1,14]. For example, a greater increase in ionized calcium was demonstrated after ingestion of fortified juice than with calcium citrate taken with a meal or with a dairy product meal in a randomized clinical trial of 10 women with normal renal function, while in the Multi-Ethnic Study of Atherosclerosis, calcium supplement use (relative risk 1.22; 95% confidence interval: 1.07–1.39) but not total calcium intake was associated with increased risk of subsequent development of coronary artery calcification [15,16]. Although it is counter-intuitive that prescribed calcium salts would lower serum calcium, the available literature suggests their association with serum calcium is surprisingly small. For example, in comparing the impact of calcium-based phosphate binders versus non-calcium-based binders on serum biochemistries, Wang et al. found in a meta-analysis of 18 clinical trials that despite a mean prescribed elemental calcium dose of 3.79 g/day, serum calcium was higher by only 0.24 mmol/L in those taking calcium-based phosphate binders [5]. the authors did not comment on a dose effect of calcium-based phosphate binders, and assessments of dietary calcium were not mentioned. If replicated, the findings of opposite effects of prescribed versus dietary sources of calcium on serum calcium levels and FGF23 could influence future studies of calcium-based phosphate binders use and dietary recommendations in CKD. 

Limitations: The usual restrictions of a cross-sectional study apply. Although large enough to find clinically relevant associations and answer our study objectives, our sample size of 81 was relatively small, and limited the precision of our estimates and ability to detect other important associations. We examined only carboxyl-terminal FGF23, and it is possible different results would have been obtained had an intact FGF23 assay been used instead. We are unable to speculate if and how the results may have changed in such a scenario. However, carboxyl-terminal FGF23 possesses a strong association with mortality, and represents a valid biomarker [2]. We obtained only one measurement of serum calcium and cFGF23 per patient, and any potential influence of variations in their levels could not be accounted for. A selection bias in which those with hypercalcemia were taken off or put on lower doses of a calcium-based phosphate binders cannot be ruled out. However, this was mitigated by calculating mean daily prescribed calcium amounts based on all prescriptions for 3 months before study enrollment. Also, we did not measure adherence to prescribed calcium-based phosphate binders intake, and can make no conclusions about actual prescribed calcium intake. Finally, we did not examine other potential determinants of calcium or FGF23—such as dialysate calcium concentrations (which were rarely adjusted in our dialysis population), 1,25-OH vitamin D levels, or residual renal function. However, the combination of dietary, clinical, biochemical, and demographic variables that we did adjust for when assessing the relationship between, and the determinants of, serum calcium and FGF23 was novel.

## 5. Conclusions

In this report, we confirmed a significant association between serum calcium and FGF23 in a multi-ethnic cohort of stable prevalent hemodialysis patients. We found differing, sometimes opposing, associations between serum calcium and FGF23 levels when considering prescribed versus dietary sources of calcium. Confirmation of these findings could influence future studies concerned with understanding the biological effects of calcium-based phosphate binders versus dietary calcium on serum calcium and FGF23 concentrations in patients receiving hemodialysis. 

## Figures and Tables

**Table 1:** Baseline characteristics.

Characteristic	
Age, mean ± SD, years	58.28 ± 14.09
Female, number (%)	30 (37%)
History of diabetes mellitus, number (%)	31 (38%)
Dialysis vintage, mean ± SD, years	2.51 ± 2.37
Dry weight, mean ± SD, kg	71.97 ± 17.37
Ethnicity, number (%) White South Asian East Asian Black	16 (20%) 24 (30%) 28 (35%) 12 (15%)
Serum cFGF23, median (IQR)[range], RU/mL	2396 (586 to 5896) [468 to 49,479]
Natural log of FGF23	7.64 ± 1.44
Serum calcium, mean ± SD, mmol/L	2.40 ± 0.18
Serum phosphate, mean ± SD, mmol/L	1.60 ± 0.50
Serum parathyroid hormone, median (IQR)[range], pmol/L	34.30 (21.30 to 79.80) [1 to 363]
Serum albumin, mean ± SD, g/L	33.83 ± 3.32
Serum 25-OH vitamin D, median (IQR)[range], nmol/L Serum hemoglobin, mean ± SD, mg/L Serum ferritin, median (IQR)[range], mcg/L Transferrin saturation, median (IQR)[range], %	39 (25 to 56) [10 to 128] 114 ± 12.3 95.5 (40 to 346) [2 to 1097] 22 (15 to 31) [6 to 78]
Using calcium-based phosphate binder, number (%)	59 (73%)
Prescribed CaCO_3_ dose, median (IQR)[range], mg/day	1500 (800 to 2500) [0 to 6250]
Prescribed activated vitamin D dose, median (IQR)[range], mcg/day	0.43 (0.13 to 0.57) [0 to 1.71]
Taking supplemental vitamin D, number (%)	19 (24%)
Dietary calcium, median (IQR)[range], mg/day	424.09 (301.13 to 626.12) [14.58 to 1037.04]
Dietary phosphate, median (IQR)[range], mg/day	831.09 (606.84 to 1026.69) [337.90 to 1645.16]
Dietary vitamin D, median (IQR)[range], mcg/day	1.66 (0.81 to 2.84) [0 to 22.74]
Dietary protein, median (IQR)[range], g/day	66.35 (53.01 to 92.85) [25.90 to 145.83]

**Table 2:** Bivariable associations of serum calcium.

Variable	Coefficient (95% CI)	*p*-Value	Adjusted *r*^2^ If Significant (%)
Natural log of FGF23	0.04 (0.01 to 0.06)	0.007	7.8
Female	0.09 (0.01 to 0.17)	0.037	4.2
Non-black race	0.10 (−0.01 to 0.21)	0.064	
Parathyroid hormone category	0.04 (0.00 to 0.08)	0.071	
Age, years	0.00 (0.00 to 0.01)	0.119	
Prescribed CaCO_3_, 1000 mg/day	−0.02 (−0.05 to 0.01)	0.157	
Dry weight, kg	0.00 (0.00 to 0.00)	0.2	
Activated vitamin D, mcg/day	0.07 (−0.04 to 0.18)	0.208	
Caloric intake, kcal/day	0.00 (0.00 to 0.00)	0.255	
Dietary calcium, 1000 mg/day	0.10 (−0.09 to 0.30)	0.302	
Serum 25-OH vitamin D, nmol/L	0.03 (−0.04 to 0.11)	0.368	
Natural log of transferrin saturation	−0.03 (−0.12 to 0.05)	0.419	
Natural log of dietary vitamin D	0.00 (−0.02 to 0.01)	0.445	
Use of a phosphate binder	0.02 (−0.04 to 0.09)	0.462	
HD vintage, year	0.01 (−0.01 to 0.02)	0.494	
Dietary protein, g/day	0.00 (0.00 to 0.00)	0.508	
Dietary phosphate, 1000 mg/day	0.04 (−0.10 to 0.19)	0.552	
Serum phosphate, mmol/L	0.01 (−0.007 to 0.09)	0.755	
Vitamin D supplementation	0.01 (−0.08 to 0.11)	0.756	
Albumin, mg/L	0.00 (−0.01 to 0.01)	0.827	
Natural log of serum ferritin	0.00 (−0.03 to 0.03)	0.909	
Serum hemoglobin, mg/L	0.00 (0.00 to 0.00)	0.97	

Parathyroid hormone categorized as less than 10, 10–49, 50–149, 150–249, or ≥250 pmol/L.

**Table 3:** Bivariable associations of the natural log of FGF23.

Variable	Coefficient (95% CI)	*p*-Value	Adjusted *r*^2^ If Significant (%)
Serum phosphate, mmol/L	1.81 (1.17 to 2.45)	<0.001	28.5
Serum hemoglobin, mg/L	−0.04 (−0.07 to −0.02)	0.002	11.3
Serum calcium, mmol/L	2.42 (0.67 to 4.16)	0.007	7.8
Parathyroid hormone category	0.37 (0.03 to 0.70)	0.031	4.7
Dietary calcium, 1000 mg/day	−1.60 (−3.16 to −0.05)	0.044	4.6
Natural log of serum ferritin	−0.23 (−0.46 to 0.01)	0.059	
Prescribed CaCO_3_, 1000 mg/day	0.22 (−0.03 to 0.47)	0.08	
Activated vitamin D, mcg/day	0.79 (−0.12 to 1.70)	0.089	
Natural log of dietary vitamin D	−0.22 (−0.54 to 0.11)	0.192	
Age, years	−0.02 (−0.04 to 0.01)	0.211	
Albumin, mg/L	−0.06 (−0.16 to 0.04)	0.217	
Dry weight, kg	0.01 (−0.01 to 0.03)	0.341	
Caloric intake, kcal/day	0.00 (0.00 to 0.00)	0.448	
Non-black race	0.30 (−0.61 to 1.20)	0.517	
Use of a phosphate binder	0.16 (−0.36 to 0.67)	0.554	
Natural log of transferrin saturation	−0.15 (−0.86 to 0.52)	0.654	
Dietary protein, g/day	0.00 (−0.01 to 0.02)	0.656	
Vitamin D supplementation	−0.15 (−0.94 to 0.64)	0.706	
HD vintage, years	−0.03 (−0.20 to 0.14)	0.743	
Natural log of serum 25-OH vitamin D	−0.09 (−0.72 to 0.54)	0.772	
Dietary phosphate, 1000 mg/day	0.14 (−1.05 to 1.34)	0.81	
Female	0.01 (−0.67 to 0.68)	0.985	

Parathyroid hormone categorized as less than 10, 10–49, 50–149, 150–249, or ≥250 pmol/L.

**Table 4:** Simple multivariable model to predict serum calcium (adjusted *r*^2^ = 22%).

Variable	Coefficient ± SE	ß Coefficient	*p*-Value
Natural log of FGF23	0.05 ± 0.01	0.39	0.002
Prescribed CaCO_3_, per 1000 mg/day	−0.03 ± 0.01	−0.27	0.021
Non-black race	0.10 ± 0.05	0.24	0.042
Age, per year	0.00 ± 0.00	0.10	0.394
Female	0.03 ± 0.04	0.08	0.479
Parathyroid hormone category	0.00 ± 0.02	0.01	0.951

Parathyroid hormone categorized as less than 10, 10–49, 50–149, 150–249, or ≥250 pmol/L.

**Table 5:** Simple multivariable model to predict natural log of FGF23 (adjusted *r*^2^ = 46%).

Variable	Coefficient ± SE	ß Coefficient	*p*-Value
Serum phosphate, per mmol/L	1.42 ± 0.33	0.43	<0.001
Serum calcium, per mmol/L	2.22 ± 0.84	0.25	0.011
Natural log of serum ferritin	−0.23 ± 0.10	–0.22	0.02
Serum hemoglobin, per mg/L	−0.02 ± 0.01	–0.22	0.023
Dietary CaCO_3_, per 1000 mg/day	−1.32 ± 0.62	–0.20	0.038
Parathyroid hormone category	0.07 ± 0.15	0.04	0.661

Parathyroid hormone categorized as less than 10, 10–49, 50–149, 150–249, or ≥250 pmol/L.

**Table 6:** Hand-built multivariable model to predict serum calcium (adjusted *r*^2^ = 38%).

Variable	Coefficient ± SE	ß Coefficient	*p*-Value
Natural log of FGF23	0.05 ± 0.01	0.46	0.001
Prescribed CaCO_3_, per 1000 mg/day	−0.04 ± 0.01	−0.35	0.005
Dietary calcium, per 1000 mg/day	0.27 ± 0.12	0.35	0.032
Dietary phosphate, per 1000 mg/day	−0.26 ± 0.14	−0.47	0.084
Dietary protein, per g/day	0.00 ± 0.00	0.62	0.008
Non-black race	0.05 ± 0.05	0.14	0.282
Dry weight, per kg	0.00 ± 0.00	−0.18	0.137
Serum hemoglobin, per g/L	0.00 ± 0.00	0.10	0.429

* Incorporating results from bivariable analyses, variables as determined by literature review, optimized using Akaike information criteria.

**Table 7:** Hand-built multivariable model to predict natural log of FGF23 (adjusted *r*^2^ = 69%).

Variable	Coefficient ± SE	ß Coefficient	*p*-Value
Serum phosphate, per mmol/L	1.77 ± 0.30	0.54	<0.001
Natural log of serum ferritin	−0.33 ± 0.08	−0.34	<0.001
Serum calcium, per mmol/L	2.18 ± 0.80	0.24	0.01
Dietary calcium, per 1000 mg/day	−1.53 ± 0.65	−0.24	0.023
Age, per year	−0.02 ± 0.01	−0.19	0.039
Serum hemoglobin, per mg/L	−0.02 ± 0.01	−0.18	0.049
Prescribed CaCO_3_, per 1000 mg/day	0.17 ± 0.09	0.17	0.071
Dietary phosphate, per 1000 mg/day	0.74 ± 0.47	0.16	0.118
Albumin, per g/L	−0.05 ± 0.05	−0.11	0.26

* Incorporating results from bivariable analyses, variables as determined by literature review, optimized using Akaike information criteria.
